# Hybrid weakness and continuous flowering caused by compound expression of FTLs in *Chrysanthemum morifolium* × *Leucanthemum paludosum* intergeneric hybridization

**DOI:** 10.3389/fpls.2023.1120820

**Published:** 2023-01-26

**Authors:** Zixuan Li, Chenyuan Mao, Xinyi Wu, Haoqing Zhou, Kunkun Zhao, Jiafu Jiang, Sumei Chen, Weimin Fang, Zhiyong Guan, Jing Zhang, Yuan Liao, Zhenxing Wang, Fadi Chen, Haibin Wang

**Affiliations:** State Key Laboratory of Crop Genetics and Germplasm Enhancement, Key Laboratory of Landscaping, Ministry of Agriculture and Rural Affairs, Key Laboratory of Biology of Ornamental Plants in East China, National Forestry and Grassland Administration, College of Horticulture, Nanjing Agricultural University, Nanjing, China

**Keywords:** hybridization, chrysanthemum, FTLs, hybrid weakness, continuous flowering

## Abstract

Hybridization is an important evolutionary mechanism ubiquitous to plants. Previous studies have shown that hybrid polyploidization of cultivated chrysanthemum, ‘Zhongshanzigui’, and *Leucanthemum paludosum* exhibit spring-flowering traits. This study explores the function of the LpFTLs gene via the phenotype of A. thaliana after heterologous transformation of the LpFTLs gene, and analyzes the mechanism ofthe continuous flowering phenotype and heterosis of hybrid offspring. The results suggest that the flowering phenotype of hybrid offspring in spring may be related to the expression of the *LpFTLs* gene. Ectopic expression of *Leucanthemum paludosumLpFTLs* in *Arabidopsis thaliana* resulted in earlier flowering, indicating that the *LpFTLs* gene also affects the flowering time in *L. paludosum.* Compound expression of *FTLs* in *C. morifolium* × *L. paludosum* intergeneric hybridization directly leads to serious heterosis in the hybrid offspring. Moreover, continuous flowering appears to be accompanied by hybrid weakness under the balance of vegetative and reproductive growth. Therefore, in future studies on chrysanthemum breeding, a suitable balance point must be established to ensure the target flowering time under normal growth.

## 1 Introduction

Floricultural crops are an integral part of our lives, with uses ranging from aesthetics to food and medicine. Seasonal flowering is an established pattern of floriculture crops, in which flowering occurs in a specific season each year. *Chrysanthemum morifolium* is one of the four most important ornamental species in cut-flowers worldwide, and its diversity with respect to growth habit, inflorescence form, and color ensures its economic importance, not only for its ornamental value but also for extensive applications in people’s daily lives (Higuchi and Hisamatsu, 2015; Bernardini et al., 2022). *C. morifolium* is a qualitative short-day flowering plant and there is no spring flowering in the first half of the year under natural production conditions. To maintain annual production, the flowering of *C. morifolium* should be controlled by photoperiod using lights, which may result in wasting energy (Fukai, 2014; Kang et al., 2019).

Although there are a variety of technical improvements, hybridization breeding remains the most effective way to develop new varieties. Hybridization is an important source of genetic variation and functional evolution and is presently recognized as having extensive evolutionary and ecological implications for promoting abundance, distribution, and rapid adaptive evolution (Barton, 2001; Paun et al, 2007). Increasing evidence indicates that the evolution of plant species is an ongoing, dynamic process that originated multiple times through hybridization and polyploidization (Soltis et al, 1993; Soltis and Soltis, 1999). In many species, hybrid vigor of the mature plant biomass or other genetic effects commonly enhances performance relative to their parents, an important factor for increasing the production of major crops such as wheat, maize, rice, soybean, and canola (Chen, 2010). Genetic models explaining the increased yield of hybrids consider the interaction of alleles at many loci, generating altered expression levels and patterns of gene expression (Birchler, 2015). However, there are always exceptions; weakness or disadvantage also appears occasionally in hybrid plant breeding. Hybrid weakness refers to the phenomenon which shows developmental inferiority, such as necrotic leaves, small stature, or feeble growth (Hannah et al., 2007). Furthermore, our understanding of the molecular mechanisms underlying the hybrid weakness is limited. Previous research has established that a gene regulation mechanism is a convincing tool for hybrid weakness (Chen et al., 2014). Gene locus interactions could also cause weakness in specific hybrids, as detrimental (incompatible) genetic interactions between parental alleles, may incur a fitness cost (Zhang et al., 2016; Shiragaki et al., 2019).

*Leucanthemum paludosum* is a small perennial plant of the Asteraceae family sown in autumn and winter, grows and blooms from early spring to late spring, and can be maintained for two to three months (Wang et al., 2014). As *L. paludosum* has the potential for early flowering, we aimed to transfer related traits to achieve continuous flowering in *C. morifolium*. Although intergeneric hybridization is difficult to achieve, in our early studies, we successfully obtained intergeneric hybrids between *C. morifolium* ‘zhongshanzigui’ and *L. paludosum via* ovule rescue (Wang et al., 2014). However, the hybrid vigor was low and weak indicating a serious disadvantage.

The switch from vegetative to reproductive growth is a key event in the life cycle of plants which is triggered by a variety of both environmental cues (notably photoperiod and temperature). A change in the environmental stimulates a network of regulatory genes, one of the most important of which is *FTL*. The *FTL* gene plays a crucial role in the flowering process of higher plants and is the signal concentration point of the flower development pathway of the reproductive organs of plants. It has a crucial regulatory role in the development of flower organs, as it can integrate the signals of different flowering pathways to regulate the flowering traits and have an impact on the flowering of the composite family, previously demonstrated in studies on the flowering physiology of chrysanthemum (Fukai, 2014; Higuchi and Hisamatsu, 2015; Wang et al., 2020). The chrysanthemum genome harbors three FT-like genes, and *FTL*3 is a key regulator of photoperiodic flowering (Sun et al., 2017a). The expression of *FTL*1 and *FTL*2 was downregulated, whereas that of *FTL*3 was upregulated under short-day (SD) conditions, and heterologous expression of *CmFTL3* in the *Arabidopsis thaliana* ft mutant could rescue the mutant phenotype, meaning that *CmFTL* could compensate for the absence of FT, act as a regulator of floral transition, and respond to the photoperiod in which a similar signal network of the genes for flowering is conserved in *Arabidopsis* and *Chrysanthemum* (Oda et al., 2012a; Xiang et al., 2012; Sun et al., 2017a). It is reasonable to speculate that the *FTL* of *L. paludosum* also plays a very important role in the hybridization of *C. morifolium* ‘zhongshanzigui’ and *L. paludosum*. Nevertheless, compared with the relatively clear *FTL* gene function in chrysanthemum, the number and function of the flowering response in *L. paludosum* and the hybrid are still unclear. Therefore, in the present study, we aimed to explore the function of the *LpFTLs* gene *via* the phenotype of *A. thaliana* after heterologous transformation of the *LpFTLs* gene, and analyze the mechanism of the continuous flowering phenotype and heterosis of hybrid offspring, which could provide a reference for the purpose of flowering and the molecular mechanism of distant hybridization breeding.

## 2 Materials and methods

### 2.1 Plant materials and phenotypic observation

The conserved plant materials *C*. *morifolium* ‘Zhongshanzigui’, *L*. *paludosum*, and their F_1_ hybrids (*C. morifolium* × *L. paludosum*) were collected from the Chrysanthemum Germplasm Resource Preserving Center, Nanjing Agricultural University, China. All the plants were propagated from cutting and grown in a greenhouse under natural light. The seedling, flower bud differentiation, flowering, and flowering decay stages of all plant materials were observed and observations were recorded for at least two years. Plant height and leaf area, weak stem thickness, and survival rate (60 days after transplanting) were also collected. Significance testing was performed using SPSS 20.0 (http://www-01.ibm.com/software/cn/analytics/spss/ ).

### 2.2 *LpFTL1*, *LpFTL*and *LpFTL*isolation

Total RNA was isolated from young, fully expanded leaves of *L*. *paludosum* using the Total RNA Isolation System (Takara Bio) following the manufacturer’s instructions. From 1μL total RNA, first-strand cDNA was synthesized using random primers and SuperScript III Reverse Transcriptase (Invitrogen). PCR amplification was performed using degenerate primers (FT-F: AYACIYTIGTIATGGTIGAYCC; FT-R: CCISWYTCICKYTGRCARTT). The PCR products were cloned using the PMD19 TA cloning kit (Takara, Japan) for confirmation by DNA sequencing. RACE PCR was then used to obtain the full-length cDNA. For the 3′ RACE reaction, the first-strand cDNA was synthesized using an oligo (dT) primer incorporating the sequence of the adaptor primer, followed by nested PCR using gene-specific primer pairs and the adaptor primer. The nested PCR adaptor primer (Abridged Anchor Primer, AAP) and the Abridged Universal Amplification Primer (AUAP) provided with the 5′ RACE System kit v2.0 (Invitrogen) were used to reamplify 5′ RACE and the internal gene-specific primer pairs to isolate full-length cDNAs of LpFTL1. A neighbor-joining tree was generated using MEGA 5 software, applying the Poisson model with gamma-distributed rates and 1000 bootstrap replicates.

Owing to the high conservation of the FTL genes (amino acid sequence identity values> 90%), *LpFTL*2 and *LpFTL*3 primer sequences were designed according to *CmFTL*2 and *CmFTL*3 (Oda et al., 2012a). The PCR products were cloned using the PMD19 TA cloning kit (Takara) and sequenced. Finally, internal gene-specific primers were designed to distinguish CmFTL2, CmFTL3, LpFTL2, and LpFTL3. The primer sequences and PCR conditions are listed in **Table S1**.

### 2.3 Expression analysis of *FTL*s *in* ‘Zhongshanzigui’, *Lpaludosum* and their hybrid

A total of five day fully expanded fourth and fifth leaves harvested from three plants were collected at the seedling stage (March 5-10), flower bud differentiation (April 5-10), and flower decay (June 5-10) of *L*. *paludosum*; the seedling stage (May 5-10), flower bud differentiation (August 5-10) and flower decay (November 5–10) of *C*. *morifolium* ‘Zhongshanzigui’; the seedling stage (March 5-10), flower bud differentiation (April 5-10 and August 5-10) and flower decay (November 1-5) of *C. morifolium* × *L. paludosum.*

RNA was extracted using the Total RNA Isolation System (Takara) following the manufacturer’s instructions. Before reverse transcription, total RNA was treated with RNase-free DNase I (Takara) at 37°C for 30 min to avoid genomic DNA contamination. For each sample, first-strand cDNA was synthesized using random primers and SuperScript III Reverse Transcriptase (Invitrogen) according to the manufacturer’s instructions. Quantitative real-time PCR was performed using a Mastercycler ep realplex 2 S system (Eppendorf, Germany). The cDNA was amplified using SYBR^®^ Premix Ex TaqTM II (Takara, Japan). PCR conditions were 95°C for 2 min, followed by 40 cycles of 95°C for 30 s, 55-58°C for 30 s, and 72°C for 30 s. Expression levels of genes were calculated relative to *EF1α* genes using the comparative quantification analysis. The relative transcript abundance was calculated using the 2−△△Ct method. The results are presented as the mean ± SD of three replicates. The primer sequences are listed in **Table S1.**

### 2.4 *Arabidopsis thaliana* transformation functional verification

The restriction sites of *Sal* I and *Not* I were added to the ends of *LpFTL1* and *LpFTL3*, respectively. After double restriction digestion, T4 ligase was used to bind to the pENTR1A entry vector. The pENTR1A-Lp*FTL*1 and pENTR1A-Lp*FTL*3 entry vectors were then recombined with the pMDC43 vector using LR recombination technology to obtain pMDC43-Lp*FTL*1 and pMDC43-Lp*FTL*3 expression vectors.

*A. thaliana* (Col-0) was transformed using the floral dip method. The seeds of the T1 generation were screened and cultured on 1/2 MS + 25 mg/L Hyg plate medium, the T3 generation was obtained by self-pollination, and the zygosity of the transgene was identified using an RT-PCR assay. Then, the T3 generation seeds and the wild type (Col-0) were cultured for 3–4 weeks in a short-day light incubator (23°C/8 h light, 18°C/16 h dark, 75%-80% relative humidity) and transferred to long-day light with the conditions of 23°C/16 h light, 18°C/8 h dark. The transgenic process was repeated twice (codes C and B) to get sufficient transgenic lines. After bolting, statistical analysis was performed every two days. Photographs were taken when 60% of the transgenic lines bloomed. The data were statistically analyzed using Excel and SPSS software.

## 3 Results

### 3.1 Continuous flowering and hybrid growth weakness of *C morifolium* × *L paludosum*

**Figure 1:**
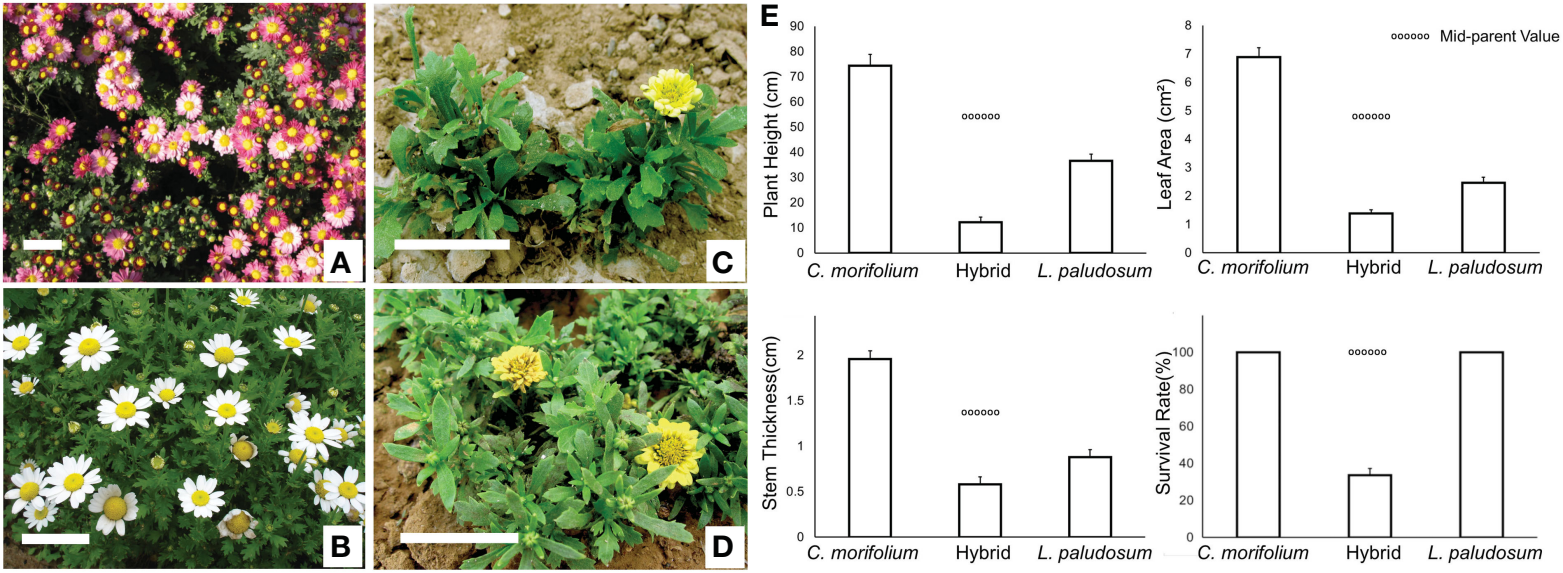
Abnormal spring flowering phenomenon and hybrid disadvantage of (*C*) *morifolium* × *L. paludosum.* Florescence state of (*C*) *morifolium* **(A)**, *L. paludosum* **(B)** and (*C*) *morifolium* × *L. paludosum* bloomed from April to May **(C)** and late October to early November **(D)** under natural conditions without any treatment. Plant height and leaf area, weak stem thickness, and survival rate of hybrids **(E)**. Values are mean ± SD. The dotted line denotes the mid-parent value. Bar=5cm.

The full flowering periods of *C. morifolium* and *L. paludosum* occurred from late October to early November and late April to May, respectively. The plants of *C. morifolium* × *L. paludosum* bloomed from late April to May and late October to early November under natural conditions without any treatment (**Figure S1**). This means that the hybrids of *C. morifolium* × *L. paludosum* flowered twice a year, from spring to early summer and autumn to winter which shown a superimposition of parental flowering patterns. It is emphasized that the state of *C. morifolium* (**Figure 1A**) and *L. paludosum* (**Figure 1B**) was natural while only a few flowers can bloom normally in spring for two years but are accompanied by a large number of poorly developed flower buds that cannot open in the later stage (**Figures 1C, D**). In addition to flowering traits, the ornamental traits of the hybrid were weaker than those of their parents. The main performance criteria consisted of decreased plant height and leaf area, slow growth, weak stem thickness, and low survival rate, which showed strong hybrid weakness (**Figure 1E**).

### 3.2 Sequences analysis of FT group genes

Three FT-like genes have been successfully cloned. The full-length sequences of *LpFTL1*, *LpFTL2*, and *LpFTL3* cDNA contained a total of 522 nucleotides. *LpFTL1*, *LpFTL2*, and *LpFTL3* are predicted to encode 174 polypeptide residues. The predicted LpFTL1, LpFTL2, and LpFTL3 protein had calculated molecular mass of 19.76 kDa, 19.79 kDa, and 19.62 kDa, and theoretical pI of 9.14, 8.07, and 7.44, respectively.

**Figure 2:**
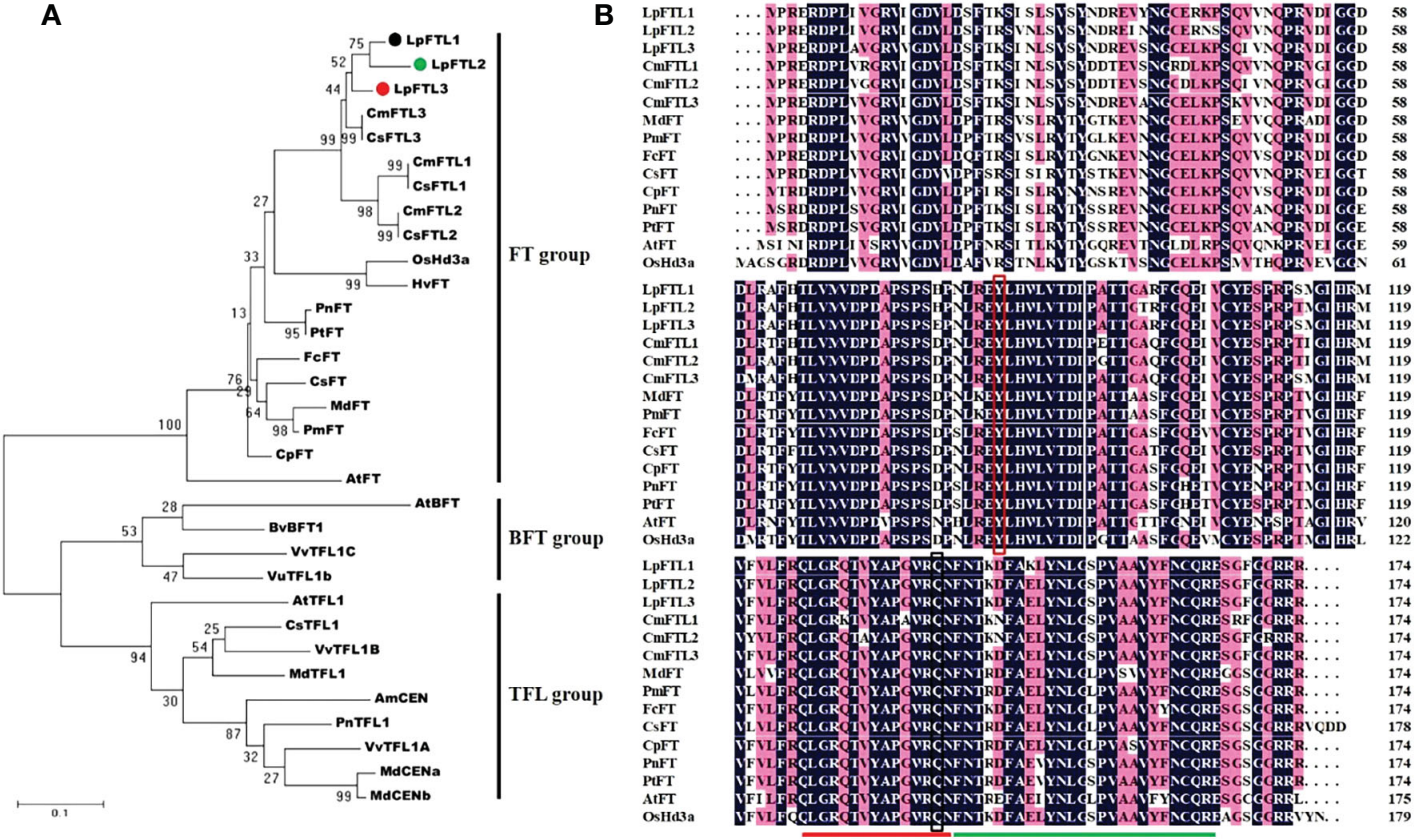
The sequences of LpFTL1, LpFTL2, and LpFTL3. **(A)** Phylogenetic tree of the deduced amino acid sequences of LpFTLs and other plant species FT sequences. **(B)** Comparison of the predicted amino acid sequences of *L. paludosum* FTLs and related proteins. Y85 (shown in red box) and Q140 (shown in black box) are essential for the formation of an external loop. The red and green lines highlight the conserved segments, required for FT-like activity.

The sequences of several other FT orthologs were used to construct a phylogenetic tree (maximum likelihood method) for the FT group genes (**Figure 2A**). The phylogenetic tree was grouped into three major clades: FT, BFT, and TFL1. As expected, LpFTL1, LpFTL2, and LpFTL3 were classified in the FT clade. It was more closely related to CmFTL3/CsFTL3 than to the others. As shown in **Figure 2B**, these three sequences encoded residues for Y85 (shown in red box) and Q140 (shown in black box), which are required for the formation of an external loop, and the conserved segments (indicated by red and green lines)

### 3.3 Expression patterns of *FTL*s in *C morifolium* ‘Zhongshanzigui’, *L paludosum* and *C morifolium* × *L paludosum*

**Figure 3:**
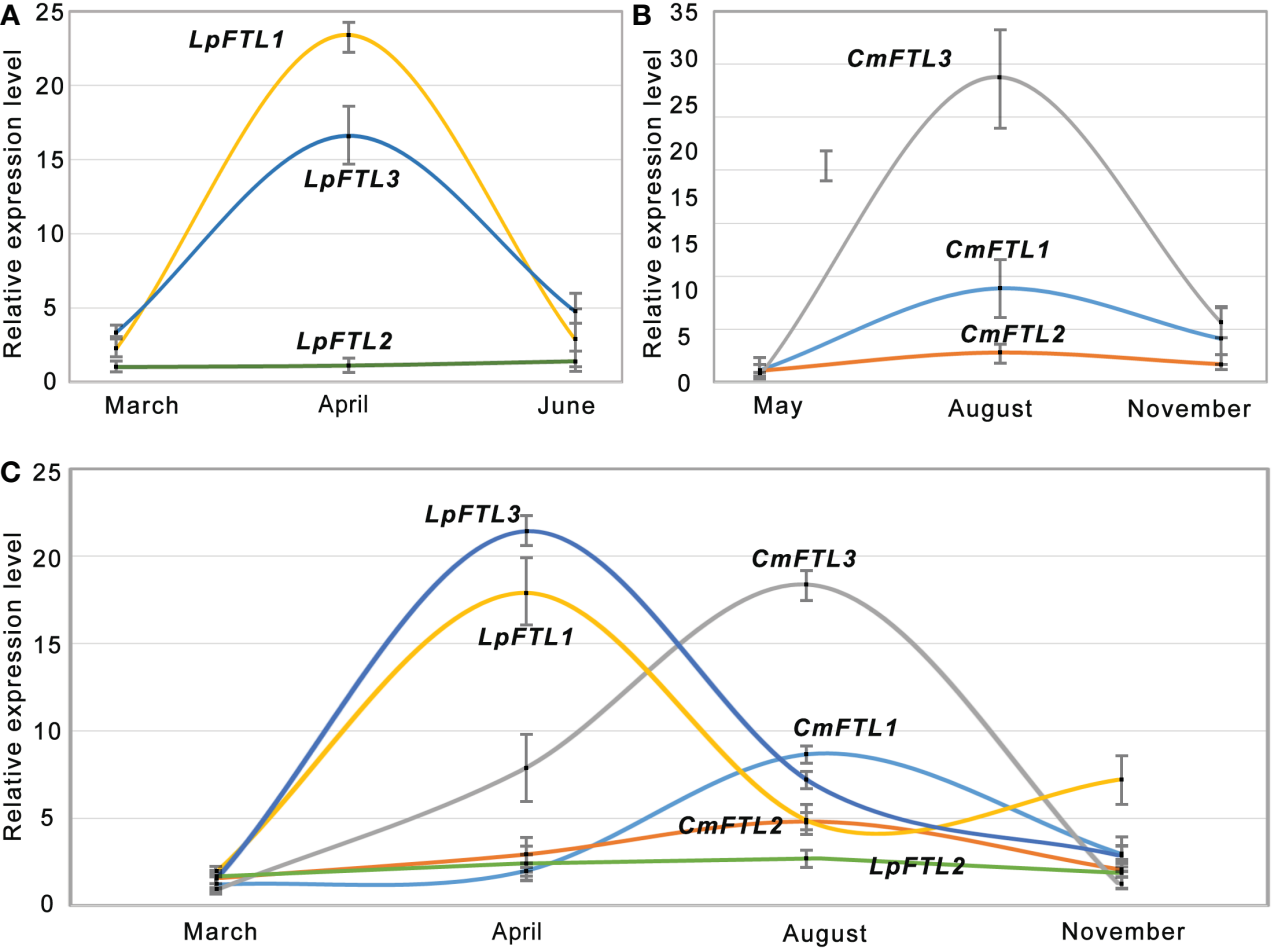
qRT-PCR verification of *FTL* genes. qRT-PCR analysis of *LpFTL1*, *LpFTL2*, and *LpFTL3* in *L. paludosum* **(A)**, *CmFTL1*, *CmFTL2*, and *CmFTL3* in *C. morifolium* **(B)** and six *FTLs* in *C. morifolium* × *L. paludosum* **(C)**.

qRT-PCR was used to examine the expression patterns of *FTLs*. The *LpFTL1* and *LpFTL3* transcripts mainly accumulated during the flower bud differentiation (April), were 15.5 and 22.1 times of the seedling stage (March), respectively, and showed a sudden decrease at the flower decay stage (June) (**Figure 3A**). The expression levels of *CmFTL1*, *CmFTL2*, and *CmFTL3* in ‘Zhongshanzigui’ are shown in **Figure 3B**. Three CmFTLs were expressed at low levels in the seedling (May) and flower decay (November) stages. The *CmFTL3* was strongly transcribed with a 29.2 value at the key flowering gene than the leaf collected at seedling and flower bud differentiation stages (August).

Although hybridization may affect gene expression to a certain extent, the expression of all *FTLs* tended to be silent during the seedling stage (March). During flower bud differentiation (April and August), the potential key flowering genes, *LpFTL1*/*LpFTL3* and *CmFTL3* transcripts, significantly increased in April and August, respectively, showing an overlay of expression modes in *C. morifolium* × *L. paludosum* (**Figure 3C**).

### 3.4 Overexpression of the *LpFTL* and *LpFTL* accelerated flowering in *Arabidopsis*

**Figure 4:**
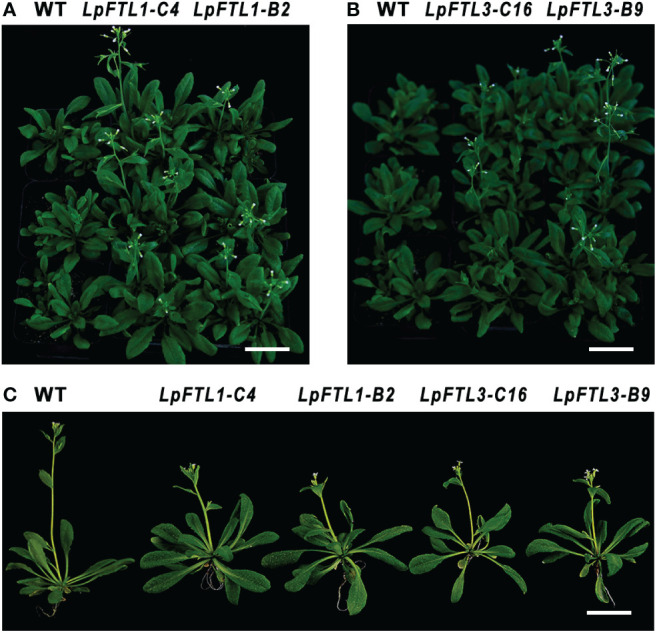
Appearance of 36-day-old wild-type WT, FTL1-C4, FTL1-B2 **(A)** and FTL3-C16, FTL3-B9 **(B)**. Florescence state of transgenic Arabidopsis line **(C)**. Bar=3cm.

In the present study, a total of 26 and 30 independent T_3_ *Arabidopsis* lines constitutively expressing *LpFTL1* and *LpFTL3* were produced, and two representative Ox lines of each gene were selected from the T3 generation seeds of transgenic *A. thaliana* to observe for expression of phenotypic effect in *LpFTL1* (**Figure 4A**) and LpFTL3 (**Figure 4B**) for *Arabidopsis*. Among these, FTL1-C4, FTL1-B2 Ox, FTL3-C16, and FTL3-B9 in multiple independent transgenic *Arabidopsis* calls showed earlier bolting and flowering than WT (Col-0) plants. The most intuitive reason is the timing of transgenic lines showing bolting or even flowering; WT Arabidopsis was still in vegetative growth and the phyllotaxy at bolting remained the same. While the number of rosette leaves was not different at flowering stage (WT VS. transgenic lines), ranging from 12 to 15 (**Figure 4C**). This result shows that *LpFTL1 andLpFTL3* are involved in controlling flowering in *Arabidopsis*.

**Figure 5:**
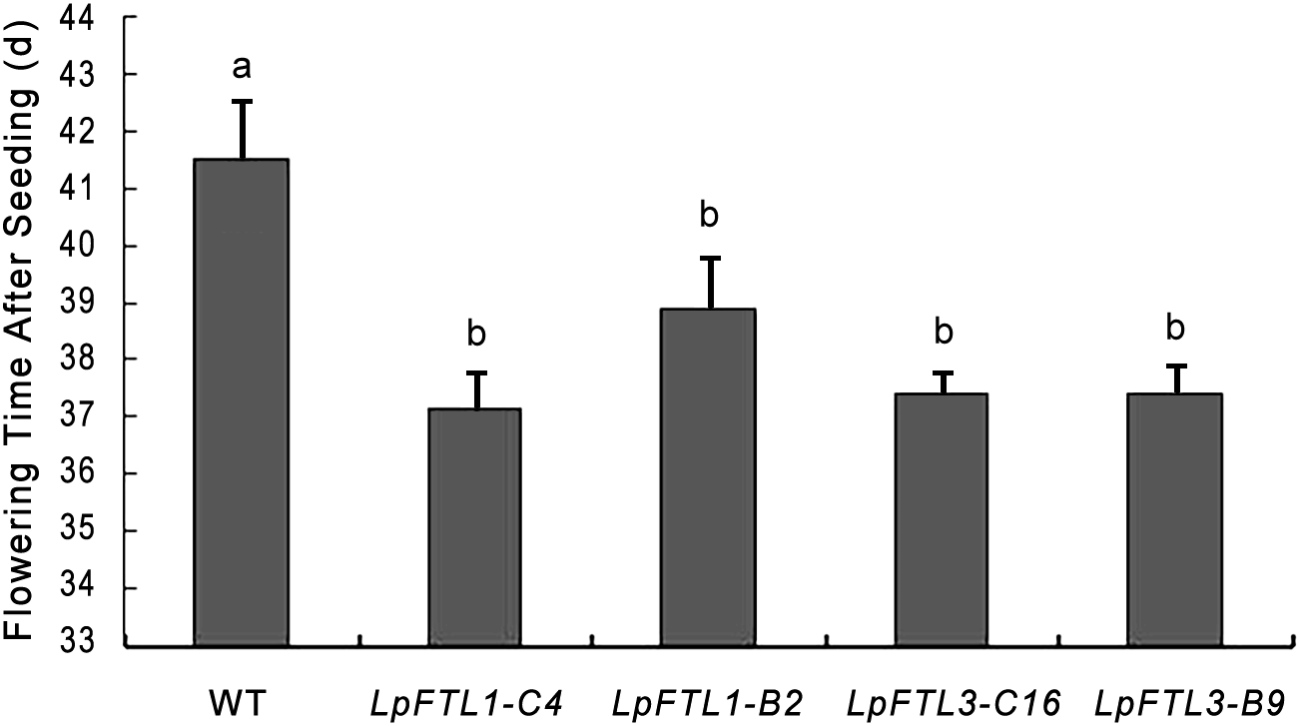
Flowering time statistics of FTL1-C4, FTL1-B2, FTL3-C16, FTL3-B9. Different superscripts indicate significant differences between means at P<0.01 according to Tukey’s test. Values are mean ± SD (from three biological replications).

Statistical analysis showed that the average flowering days from the sowing of *LpFTL1* ox were 37.2 d and 38.9 d and those from the sowing of *LpFTL3* ox were 37.4 d and 37.4 d, while that of control WT *Arabidopsis* was 41.50 d; the difference was more than 2.5 d, above the significance level of 0.05. The mean flowering days of the transgenic plants were significantly different from those of the control WT *Arabidopsis* (**Figure 5**).

## 4 Discussion

### 4.1 Continuous flowering of *C morifolium* × *L paludosum* intergeneric hybridization

Hybridization is an important mechanism of genetic variation and functional evolution (Richards, 2003; Kearney, 2005). New combinations of favorable genes come from different parents, potentially resulting in heterosis. In addition to the parent phenotypes, nascent hybrids often show novel traits that do not exist in the contributing parents, such as increasing levels of heat, cold, drought tolerance, pest resistance, flowering time change, and organs allowing their offspring to thrive in harsh environments (McClintock, 1983; Paun et al., 2006; Birchler, 2015; Botet and Keurentjes, 2020). In *Arabidopsis* allotetraploids, the expression of *A. thaliana* and *A. arenosa FLC* loci is additive, leading to a late-flowering phenotype (Wang et al., 2006). The flowering time directly affects plant reproduction and adaptation. In our study, the natural flowering of *C. morifolium* was in autumn, while *L. paludosum* was in the spring. Intergeneric hybrids are powerful, and the only way for Chrysanthemums to put forth florescence. Although hybrids between different genera are difficult to obtain, particularly between two species with relatively distant classifications, we obtained hybrids between *C. morifolium* and *L. paludosum*. After two years of observation, we ensured that all hybrids exhibited continuous flowering phenotype, and their flower bud differentiation in April and August, which led to flowering under natural conditions without any treatment. These results were consistent with our breeding objectives.

### 4.2 LpFTLs is a key gene to promote the continuous flowering of *C morifolium* × *L paludosum*

In the analysis of FTLs gene expression in hybrids, the results clearly showed that the FTLs gene is involved in the opening of *C. morifolium* × *L. paludosum*. In the flowering process, of *L. paludosum* and *C. morifolium* hybrids, it can be tentatively concluded that the first spring flowering is mainly affected by the *LpFTL1* and *LpFTL3* genes from *L. paludosum*, whereas the second flowering in autumn is determined by the *CmFTLs* gene from *C. morifolium*. Therefore, we speculate that the introduction of *LpFTLs* also promotes early flowering. From the results, it can be concluded that the *LpFTLs* gene from *L. paludosum* regulates flowering and the flowering period of *A. thaliana*, successfully converting *LpFTL1* and *LpFTL3* and was 3–5 days earlier than that of WT. Some studies on the verification of the transgenic function of *A. thaliana* cloned from *C. morifolium* showed that function was super-expressed by significantly promoting the early flowering of *A. thaliana* (Oda et al., 2012a; Higuchi and Hisamatsu, 2015; Bernardini et al., 2022). These results are in accordance with our study and prove that the *FTL* gene prevalent in a variety of plants plays a significant role in regulating flowering and this gene acting on reproductive growth is also similar (Storchova et al., 2015).

After the *FTL* protein was identified as an anthocyanin, a large number of researchers have isolated, identified, and functionally studied *FTL* homologous genes from different varieties of crops such as rice, wheat, fruit (apples), vegetables (tomatoes, potatoes), and ornamental crops (orchids, orange stalks, and chrysanthemums), which have a large number of early or late flower expression (Trankner et al., 2011; Li et al., 2014; Higuchi and Hisamatsu, 2015). Among the many varieties, the rational use of interspecific hybridization, intergeneral hybridization, and transgenics will create a broader possibility of flowering regulation (Chen et al., 2014; Qi et al., 2018; Mitrofanova et al., 2020). Most ornamental chrysanthemums are short-day plants, and if the *LpFTLs* gene is overexpressed in a chrysanthemum, it is likely to change its original flowering characteristics, such as the phenotype of continuous flowering in offspring. However, the exogenous *FTL* gene and the endogenous *FTL* gene may have a mutual influence. In an experiment to transfer the cotton *FTL* gene to tobacco, data suggested that adequate levels of transgenic cotton *FTL* might interfere with the balance of endogenous *FTL* inducers and inhibitors, leading to phenotypic changes in transgenic tobacco (Faure et al., 2007). Therefore, to apply the *FTL* gene to the breeding of continuous flowering, it is also necessary to study species as target plants in a more detailed and clearer model.

### 4.3 Hybrid weakness caused by continuous flowering after hybridization

Elucidating the molecular basis of the hybrid phenotype will contribute to our understanding of evolution and enhance crop breeding. In the present study, all hybrids exhibited a continuous-flowering phenotype. As chrysanthemum is produced by vegetative propagation, it can be cloned using cuttings, which could keep early flowering characteristics. However, success is temporary, and the growth characteristics show serious hybrid weakness compared to their parents. Therefore, in our later study, it is reasonable to expect a backcross between the hybrid and paternal ‘Zhongshanzigui’ for better ornamental traits.

**Figure 6:**
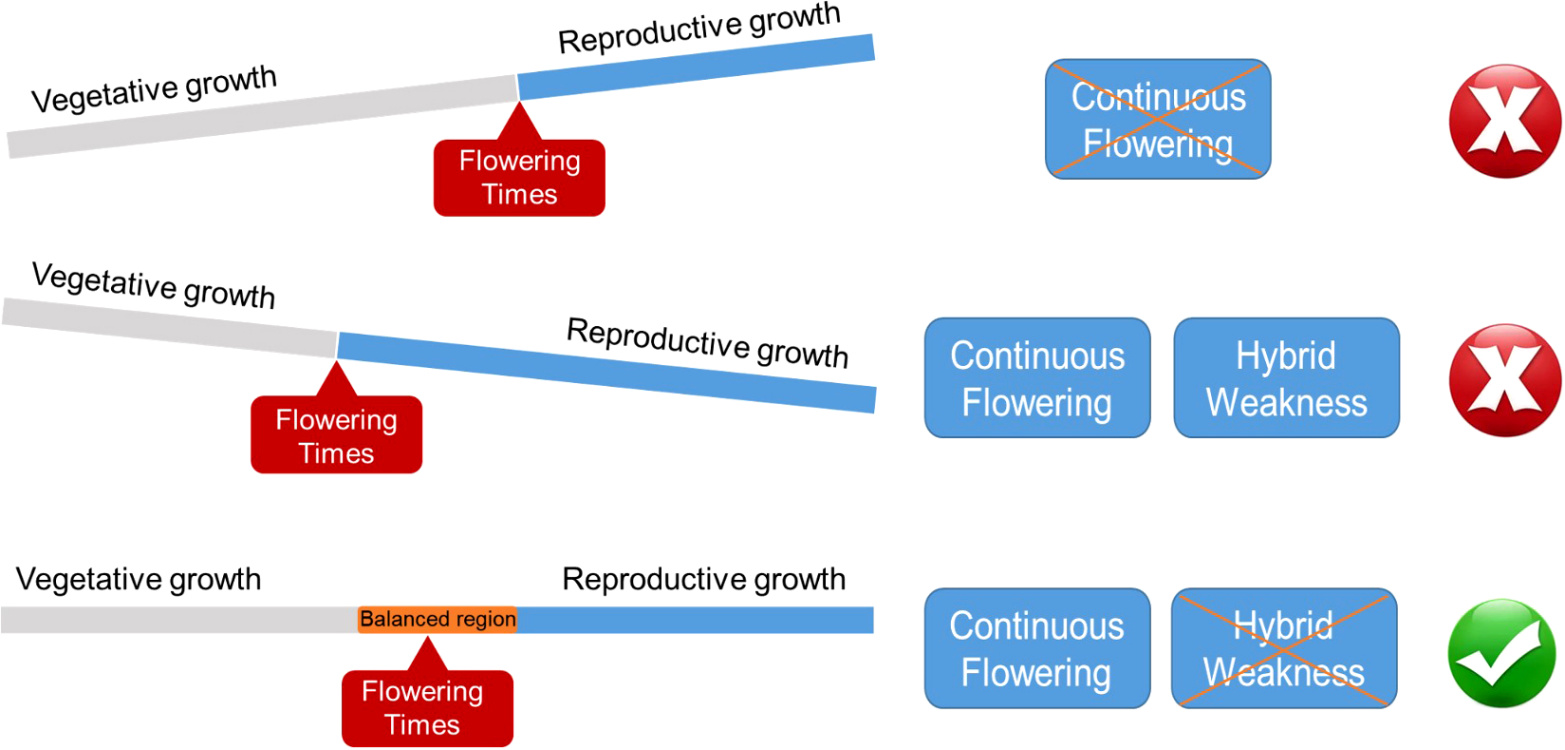
Breeding target model for continuous flowering and hybrid weakness balancing vegetative and reproductive growth. A suitable balance point is to be confirmed to ensure the target flowering time under the normal growth state.

A prevailing model explains how hybrid phenotypes in which deleterious parental alleles are compensated in hybrid progeny by cumulative enhancement of gene expression (Chen et al., 2014; Tikhenko et al., 2015). Although this matches with our aim to get continuous flowering as an improvement of ornamental traits, it was found in our research center that this is caused by compound expression leading to shorter vegetative growth, especially after the appearance of the first flowers in less than two months. This directly leads to the weakness of heterosis in hybrid offspring. Compared with other crops, continuous flowering is a fascinating goal for ornamental plants; however, strong growth and higher biomass are often ignored. Nevertheless, an intuitive model of continuous flowering observed in our present study appears to be accompanied by hybrid weakness under the balance of vegetative and reproductive growth. Therefore, in future chrysanthemum breeding programs, a suitable balance point must be confirmed to ensure the target flowering time is under normal growth (**Figure 6**).

A previous study in maize documented cases of positive associations between targeted gene expression and superior phenotypes in hybrids, which is inclusive of the occurrence of hybrid weakness because the actual results can be condition-dependent (Romagnoli et al., 1990). Meanwhile, some slow growth may be due to the haploid level of each parental genome (Sun et al, 2017b). Although the model discussed above appears to be a simple genetic model, the molecular basis of hybrid weakness is usually complex and often involves incremental effects and interactions of genes at multiple loci, most of which have not been characterized, particularly in ornamental plants (Tikhenko et al., 2015). Thus, breeding for continuous flowering either through introgression or transgenics should simultaneously target multiple loci to achieve a desired level of equilibrium (Alcazar et al., 2009). This suggests the need for further identification and molecular characterization of the weakness of hybrid loci. Such findings could facilitate the development of ornamental plants to efficiently utilize hybridization.

## Data availability statement

The original contributions presented in the study are included in the article/**Supplementary Material.** Further inquiries can be directed to the corresponding author.

## Author contributions

HW and FC contributed to conception and design of the study. HW, JZ, YL and ZW organized the data. HW, WF, SC, ZW, HZ and JJ performed the statistical analysis. HW and XW wrote the first draft of the manuscript. CM and ZL wrote sections of the manuscript. KZ, HW, SC, ZL, JJ, WF, and FC contributed to writing-review and editing. All authors contributed to the article and approved the submitted version.

## Supplementary material

Supplementary Figure 1 Flowering time of the hybrid and their parents.
