# Supplementary figures and images for: Hybrid weakness and continuous flowering caused by compound expression of FTLs in Chrysanthemum morifolium × Leucanthemum paludosum intergeneric hybridization
Source: Front Plant Sci. 2023 Jan 26;14:1120820. doi: 10.3389/fpls.2023.1120820 (PMC9911212; doi:10.3389/fpls.2023.1120820)

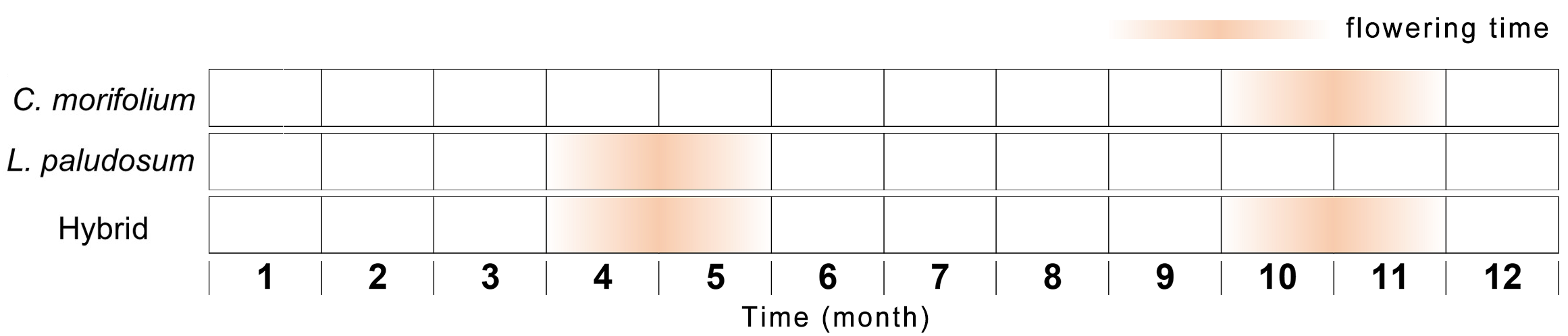

Supplement: Supplementary Figure 1 — Flowering time of the hybrid and their parents. [file Image_1.tif]
