# Supplementary material for: Hybrid weakness and continuous flowering caused by compound expression of FTLs in Chrysanthemum morifolium × Leucanthemum paludosum intergeneric hybridization
Source: Front Plant Sci. 2023 Jan 26;14:1120820. doi: 10.3389/fpls.2023.1120820 (PMC9911212; doi:10.3389/fpls.2023.1120820)
Supplement: Supplementary file 2 [file Table_1.docx]

**Sequence of *LpFTL1*/*2*/*3***

>LpFTL1

ATGCCGAGAGAAAGGGATCCATTGATTGTTGGACGTGTAATAGGCGATGTTCTTGATAGTTTCACCAAGTCTATCAGCCTCTCTGTCTCTTATAATGATAGGGAAGTGTACAACGGGTGTGAGCGAAAACCCTCTCAAGTTGTGAACCAACCGAGAGTTGATATTGGAGGTGACGATTTGCGCGCTTTTCACACATTGGTGATGGTGGATCCTGATGCTCCAAGTCCAAGTCACCCTAACTTAAGGGAATACTTGCATTGGCTGGTTACTGATATCCCAGCGACCACAGGAGCACGATTTGGTCAAGAAATCGTGTGCTATGAAAGCCCAAGACCATCAATGGGAATCCATCGCATGGTTTTTGTTCTATTCCGGCAATTGGGTCGACAAACTGTGTACGCCCCAGGATGGCGCCAGAACTTCAACACCAAAGACTTTGCGAAGCTCTACAACCTCGGATCTCCAGTAGCCGCGGTTTATTTTAACTGTCAACGCGAAAGTGGATTTGGTGGACGACGAAGATAGGATATAAGGCATGTCCATG

>LpFTL2

ATGCCGAGGGAAAGGGATCCATTGATTGTTGGACGTGTAATAGGCGATGTTCTTGATAGCTTCACCAGATCTGTCAACCTTTCTGTCTCCTATAATGATAGGGAAATTAATAACGGGTGTGAGCGAAATTCCTCTCAAGTTGTGAACCAACCCAGAGTTGATATTGGAGGTGACGATTTGCGCGCTTTTCACACATTGGTTATGGTGGATCCTGATGCTCCAAGTCCAAGTCACCCTAACTTAAGGGAATACTTGCATTGGCTGGTTACTGACATCCCAGCCACCACGGGAACACGATTTGGTCAAGAGATCGTGTGCTATGAGAGCCCAAGACCAACAATGGGAATTCATCGCATGGTTTTTGTTCTATTCCGGCAATTGGGTCGACAAACTGTGTACGCCCCAGGATGGCGCCAGAACTTCAACACCAAAGACTTCGCGGAACTCTACAATCTCGGATCTCCAGTAGCCGCGGTTTACTTTAACTGTCAACGTGAAAGTGGATTTGGTGGACGACGAAGATAAGATATATAAGGCATGTCCATGGCATGCATGCACATGACGTTACAAATGCATAT

>LpFTL 3

ATGCCGAGGGAAAGGGATCCATTAGCTGTTGGACGTGTAGTAGGCGATGTTCTTGATAGTTTCACCAAGTCTATCAACCTCTCTGTGTCCTACAATGATAGGGAAGTTAGCAACGGGTGCGAGCTAAAACCCTCTCAAATTGTCAACCAACCGAGAGTTGATATAGGAGGTGATGATCTGCGCGCTTTTCACACATTGGTGATGGTGGATCCTGATGCTCCAAGTCCTAGTGAGCCTAACTTAAGGGAATACTTGCATTGGCTGGTTACTGATATCCCAGCTACCACTGGAGCACGGTTTGGTCAAGAAATCGTGTGCTATGAGAGCCCAAGGCCATCAATGGGAATTCATCGCATGGTTTTTGTGCTATTCCGTCAATTGGGGCGACAAACTGTGTACGCCCCAGGATGGCGCCAGAACTTCAACACCAAAGACTTTGCGGAGCTCTACAACCTCGGATCTCCAGTAGCCGCGGTTTACTTTAACTGTCAACGCGAAAGTGGATTTGGTGGACGACGGAGATAAGAGTTAATGCGTGTTCATGGCATGCAGATGACGTTACAA

**qPCR primer sequence**

Left primer of qLpFTL1: GTTTCACCAAGTCTATCAG

Right primer of qLpFTL1: TTGGTGTTGAAGTTCTGGCG

Left primer of qLpFTL2: GCTTCACCAGATCTGTCAA

Right primer of qLpFTL2: TTGGTGTTGAAGTTCTGGCG

Left primer of qLpFTL3: CTATTCCGTCAATTGGGGCG

Right primer of qLpFTL3: CGTCATCTGCATGCCATGAA

Left primer of qCmFTL1: AATCGTGTGCTATGAGAGCC

Right primer of qCmFTL1: GCTTGTAACGTCCTCTTCATGC

Left primer of qCmFTL2: ATGTGTTATTCCGGCAATTGGGTCG

Right primer of qCmFTL2: AAATATGCATTTGTAACGTCATGTG

Left primer of qCmFTL3: CTATGAGAGCCCAAGGCCATCAATG

Right primer of qCmFTL3: TGATGTTCGTGCTTTCAATATGTAT

**PCR conditions:**

SYBR Premix Ex Taq Ⅱ 10.0 µl

Forward primer(10μM) 1.0 µl

Reverse primer(10μM) 1.0 µl

cDNA template 5.0 µl

ddH2O 3.0 µl

Total 20.0 µl
